# Quantitative Macromolecular Modeling Assay of Biopolymer-Based Hydrogels

**DOI:** 10.3390/gels10110676

**Published:** 2024-10-22

**Authors:** Nada Abroug, Lisa Schöbel, Aldo R. Boccaccini, Hermann Seitz

**Affiliations:** 1 Chair of Microfluidics, Faculty of Mechanical Engineering and Marine Technology, University of Rostock, 18059 Rostock, Germany; hermann.seitz@uni-rostock.de; 2 Institute of Biomaterials, Department of Materials Science and Engineering, Friedrich Alexander-University Erlangen-Nuremberg, 91058 Erlangen, Germany; lisa.schoebel@fau.de (L.S.); aldo.boccaccini@fau.de (A.R.B.); 3 Department Life, Light & Matter, Interdisciplinary Faculty, University of Rostock, 18059 Rostock, Germany

**Keywords:** rubber elasticity, swelling, biopolymer network structure, bound water, visco-elasticity

## Abstract

The rubber elasticity theory has been lengthily applied to several polymeric hydrogel substances and upgraded from idealistic models to consider imperfections in the polymer network. The theory relies solely on hyperelastic material models in order to provide a description of the elastic polymer network. While this is also applicable to polymer gels, such hydrogels are rather characterized by their water content and visco-elastic mechanical properties. In this work, we applied rubber elasticity constitutive models through hyperelastic parameter identification of hydrogels based on their stress–strain response to compression. We further performed swelling experiments and determined the intrinsic properties, i.e., density, of the specimens and their components. Additionally, we estimated their equilibrium swelling and employed it in the swelling-equilibrium theory in order to determine the polymer–solvent interaction parameter of each hydrogel with regard to cross-linking. Our results show that the average mesh size obtained from the rubber elasticity theory can be regarded as a concentration-dependent characteristic length of the hydrogel’s network and couples the non-linear elastic response to the specimens’ inherent visco-elasticity through hysteresis as a quantifier of energy dissipation under large deformation.

## 1. Introduction

Biopolymer-based hydrogels are promising materials for various applications due to their biomimetic properties [1]. These properties can be modified by means of cross-linking, which targets intermolecular interactions within components of the hydrogels, in turn affecting their macroscopic behavior. Additionally, biopolymer hydrogels are highly hydrophilic, which leads to their water-absorption and -retention capacity. This characteristic makes them susceptible to swelling when placed in a solvent. Imbibed water molecules deform the polymer network, changing the specimen’s volume as well as its mechanical response and fracture toughness. Investigating hydrogel swelling is relevant to applications where the specimen has to be immersed in a culturing medium for a long duration, e.g., in tissue engineering approaches. In such applications, mechanical performance and integrity [2] are crucial, especially when mechanical stimulation is involved.

To investigate the role of interstitial water flow in mechanical behavior, poro-elastic [3] and poro-visco-elastic [4] modeling can be applied. Both approaches are based on a linear elastic or visco-elastic characterization of the solid matrix, combined with a description of the fluid flow throughout the porous phase based on Darcy’s law. However, these approaches mostly require a computational framework or finite-element numerical simulations in order to estimate the poro-elastic parameters or the swelling and mechanical behavior of hydrogel materials.

In this study, we employ the established rubber elasticity theory in order to probe the mechanical response of biopolymer-based hydrogels in relation to their network structure and viscous phase. The theoretical approach allows us to describe the polymer network’s average structural parameters from the shear modulus of the bulk hydrogel, which provides us with a length scale of the average pore size. Rubber elasticity was implemented through direct hyperelastic parameter identification (PI), which was applied to the stress–strain response to compression with regard to swelling. The selected Neo-Hookean and Arruda–Boyce hyperelastic models were established through a statistical mechanical approach providing a macromolecular description of the strain energy density function [5,6]. While both models are based on the same simplifications and assumptions, each provides a different network chain response to macro-scale deformation of the hydrogel. We further applied the obtained parameters in the swelling-equilibrium theory [7], which provides a thermodynamical description of the configurational entropy change stemming from solvent imbibition and permeation throughout the polymer networks. This allowed us to estimate the polymer–solvent interaction parameter which relates to the hydrogels’ solubility and miscibility in solvent. We implemented empirical and intrinsic properties in our investigation, mainly the densities of the specimens and their components, as well as the change in their volumetric fractions with swelling. We aimed at coupling the hydrogels’ compositions of polymeric and viscous phases to their bulk mechanical and swelling behavior and to their network structural properties. Moreover, we investigated the obtained average pore-size estimate of the polymer network in terms of its viscous aspects and with regard to the total dissipated energy during mechanical deformation.

## 2. Results and Discussion

### 2.1. Compressive Stress–Strain Response: Hyperelastic PI

**Figure 1:**
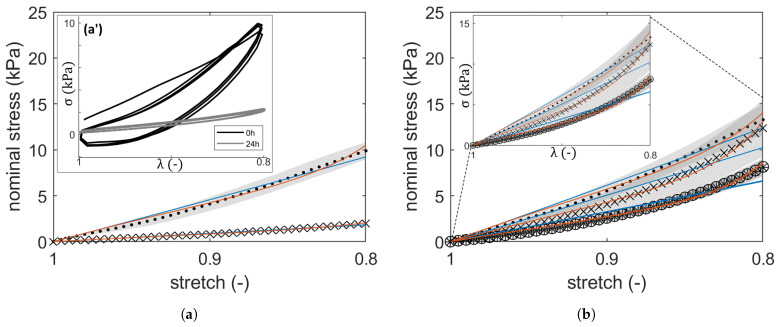

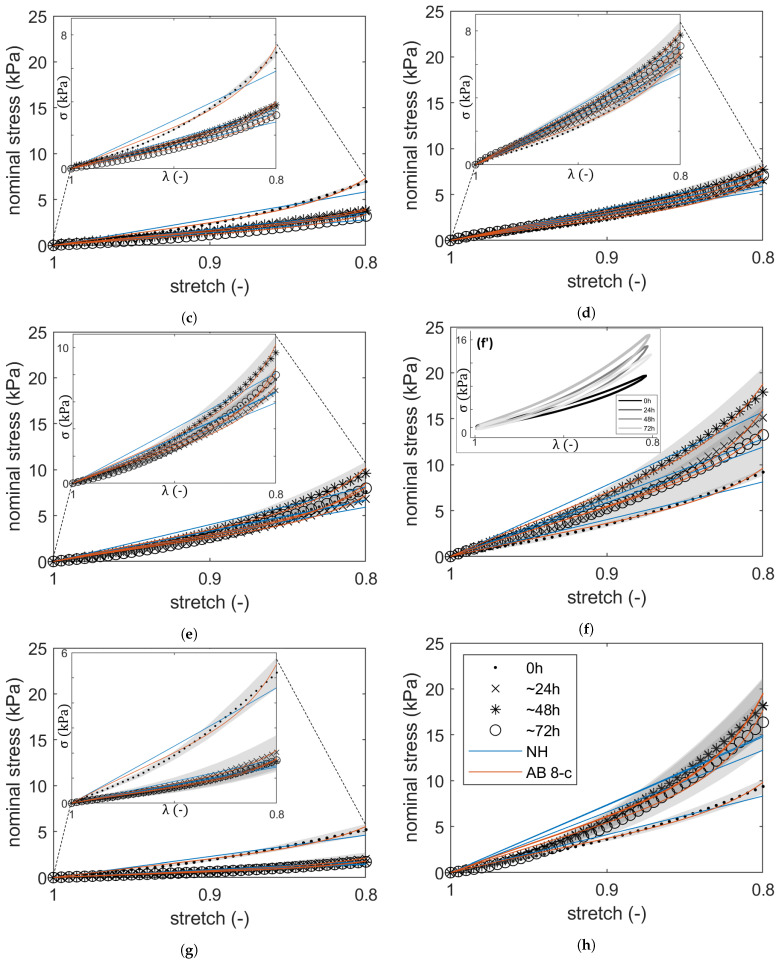
Curve-fit of the Neo-Hookean ((NH) blue) and Arruda–Boyce ((AB) red) hyperelastic models to the average loading responses of the hydrogels in their initial state (at 0 h (**·**)) and during swelling (at 24 h (×), 48 h (*) and 72 h (∘)). (**a**) algGEL_0_. (**b**) algGEL_1_. (**c**) ADAGEL_0_. (**d**) ADAGEL_1_. (**e**) ADAGEL_2_. (**f**) ADAGEL_3_. (**g**) ADAGEL_4_. (**h**) ADAGEL_5_. (**a-a’**,**f-f’**) display hysteresis loops for algGEL_0_ and ADAGEL_3_; hysteresis was observed during loading–unloading cycles for all specimens. The shaded areas represent the standard deviation (n = 3 each).

The observed hysteresis loops (such as in Figure 1(a-a’,f-f’)) during a loading–unloading cycle indicate that the scaffolds display a visco-elastic mechanical response. The mechanical response of the hydrogels in their as-prepared state seems relatively similar. Strain-stiffening can be observed (Figure 1), and is best captured using the eight-chain model. Throughout the swelling duration in 0.1 M CaCl_2_, covalently cross-linked hydrogels show an increasing compressive strength in the specimens ADAGEL_3_ and ADAGEL_5_, whereas enzymatically cross-linked ADAGEL_2_ exhibits a modest increase in stress response while swelling in ultra-pure water (UPW). algGEL_1_ and ADAGEL_1_ show a decreasing resistance to deformation and a stabilized response while placed in 0.1 M CaCl_2_ for the swelling duration. ADAGEL_4_, which was cross-linked in situ with 0.1 M CaCl_2_, exhibits a similar response to ADAGEL_0_ and algGEL_0_ when swelling in UPW.

Moduli of rigidity obtained from the fits of average stress–strain responses to Equations (2) and (4) show that $G_{\mathrm{NH}}>G_{\mathrm{AB}}$ for all hydrogels (Table A1), where $G_{\mathrm{NH}}$ describes the shear modulus according to the Neo-Hookean model and $G_{\mathrm{AB}}$ the shear modulus according to the Arruda–Boyce model.

**Figure 2:**
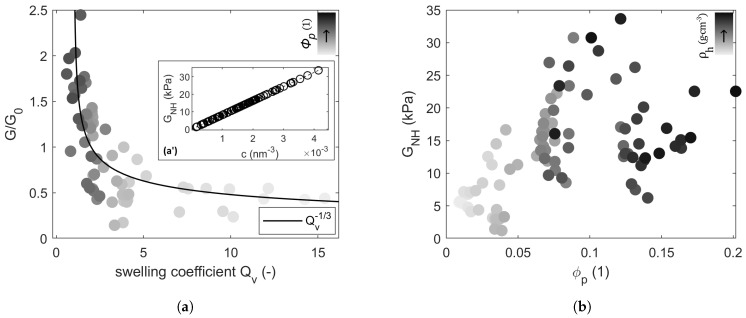
Obtained shear moduli $G_{\mathrm{NH}}$: (**a**) General scaling of $G/G_{0}$ to swelling coefficient, (**a-a’**) number of cross-linkages c=ν/2. (**b**) Variation in the polymer volumetric fraction $\phi_{p}$. The gray-scale color gradient represents (**a**) an increase in polymer fraction $\phi_{p}$ and (**b**) an increase in hydrogels’ density $\rho_{h}$.

The cross-link density obtained through the affine perfect chain model, as in Section 4.4.2, is linearly proportional to $G_{\mathrm{NH}}$ (Figure 2(a-a’)). This allows us to use shear moduli as a direct indicator of cross-linking density and, thus, in all subsequent analyses, to examine all the hydrogels jointly. $G_{\mathrm{NH}}$ shows a rather weak positive correlation to polymer volumetric fraction (Figure 2b). Shear moduli during swelling, normalized against the initial modulus $G_{0}$ of hydrogels in their as-prepared state, fulfill $G/G_{0}\approx Q_{v}^{-1/3}$ (Figure 2a), where $Q_{v}$ is the volumetric swelling coefficient.

### 2.2. Swelling Experiments

**Figure 3:**
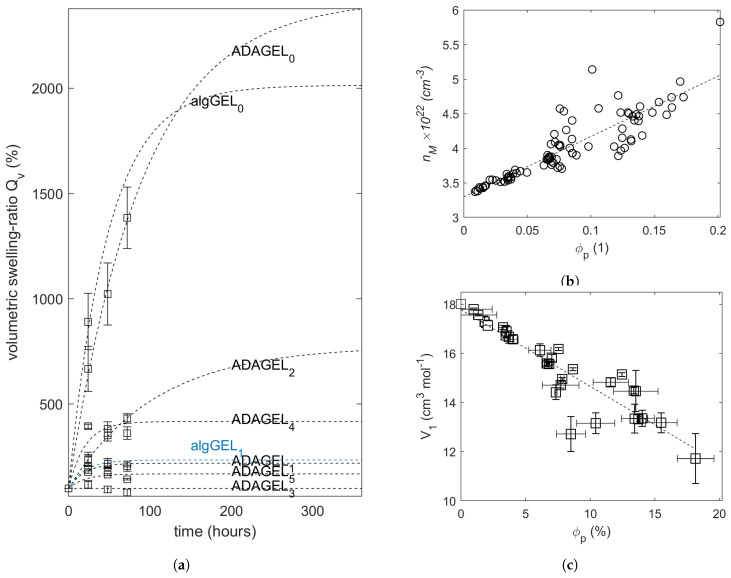
(**a**) Volumetric swelling behavior of the hydrogels (applied fit in dashed lines). Linear dependence between polymer volume fraction $\phi_{p}$, (**b**) number density of solvent molecules $n_{M}$, and, thus, (**c**) the solvent molar volume $V_{1}$. The error bars represent the standard deviation (n = 3 each).

Figure 3a shows the volumetric swelling ratio of the hydrogels after up to 72 h of swelling in UPW or 0.1 M CaCl_2_. The applied fit to first-order logarithmic swelling and the levels of equilibrium swelling are depicted. It is visible that the combination of alginate or ADA with GEL as a physical blend (algGEL_0_ and ADAGEL_0_) results in high amounts of swelling due to the uncrosslinked and thus loose state of the resulting hydrogel. For instance, all algGEL_0_ specimens fracture and disintegrate completely following 24 h of swelling in UPW. When these samples are placed in 0.1 M CaCl_2_, only negligible differences between algGEL_1_ and ADAGEL_1_ can be observed. Generally, when comparing the swelling of the hydrogels in UPW and 0.1 M CaCl_2_, it is apparent that the latter is less pronounced, and thus the effect of cross-linking on restraining swelling is obvious firsthand.

The polymer volumetric fraction $\phi_{p}$, and the average molecular number density of the bulk viscous phase $n_{M}$ are two dependent variables that were not determined separately, as provided in Section 4.2.2. $n_{M}$, and equivalently its corresponding molar volume $V_{1}$, demonstrate a linear relationship to $\phi_{p}$ (Figure 3b,c).

**Figure 4:**
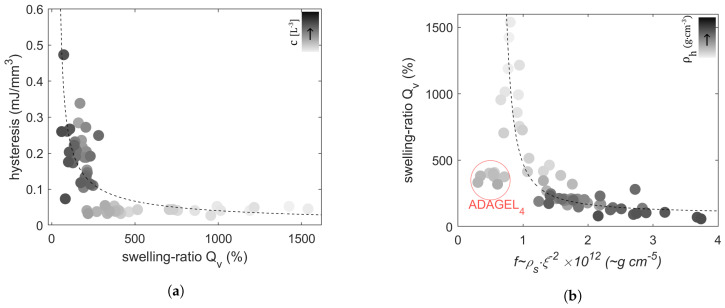

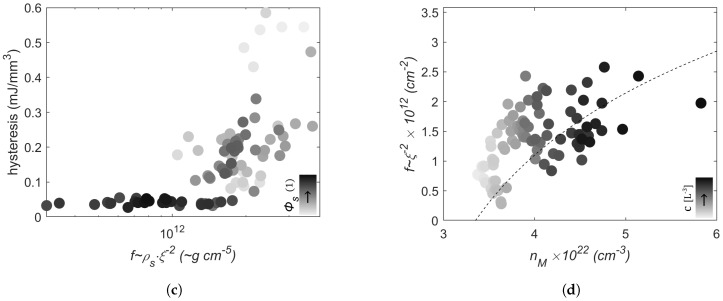
Relating hysteresis to the hydrogels’ network structural parameters. (**a**) Evolution of hysteresis with swelling ratio, (**b**) swelling ratio relation to $f\propto \rho_{s}\xi^{-2}$, (**c**) hysteresis evolution with $\log(\rho_{s}\xi^{-2})$, (**d**) dependence of f on number density of solvent molecules $n_{M}$ (fit performed for data by assuming f=0 for free water ($n_{M,\mathrm{free}\;\mathrm{water}}\approx 3.337\cdot 10^{22}{\mathrm{cm}}^{-3}$)). Gray-scale color gradient represents (**a**) and (**d**) an increase in cross-linking density *c*, (**b**) an increase in hydrogels’ density $\rho_{h}$, (**c**) an increase in water content $\phi_{s}$.

We observe an exponential decay of the obtained hysteresis over swelling ratios (Figure 4a), and the latter over the friction coefficient f (Figure 4b). We can recognize that hysteresis does not amplify with higher water content in the hydrogels and shows, rather, an exponential increase with f (Figure 4c). Furthermore, with ξ being the average mesh-size, f, depicted as $f\propto \xi^{-2}$ in order to discard the dependence between $\rho_{s}$ and $n_{M}$, shows a logarithmic trend with the solvent’s average molecular number density $n_{M}$ (Figure 4d).

In the following, Table 1, the obtained average structural parameters from the performed swelling measurements, rubber elasticity and equilibrium-swelling theories are summarized. The polymer–solvent interaction parameter χ seems to be dependent in the first place on ionic cross-linking, and increases with additional contributions from enzymatic cross-linking.

### 2.3. Discussion

Swelling experiments and large-strain compression tests were performed on biopolymer-based scaffolds. The evolution of the hydrogels’ compositions was measured, and the molar properties of the interstitial fluid were determined. Direct hyperelastic parameter identification of the Neo-Hookean and Arruda–Boyce models were performed. Both are constitutive models of the rubber elasticity theory and yield a macromolecular description of the strain energy density function. Moreover, rubber elasticity was implemented to describe the deformation of swollen polymer networks [7,8]. The obtained average network structural parameters were further employed in the equilibrium-swelling equation to determine the polymer–solvent interaction parameter χ of each specimen group. Moreover, we reviewed the obtained hysteresis from the mechanical tests in light of swelling, water content, and even the polymer–solvent friction coefficient in order to examine any dependence.

In the case of Neo-Hookean and Arruda–Boyce hyperelastic models, the invariant-based continuum mechanics provide a phenomenological description equivalent to the statistical mechanics approach [5,6]. The latter is based on the macromolecular representation of the polymer network. Moreover, the non-linear deformation is defined solely by the first invariant in both models, which makes a direct fit applicable to the estimation of materials’ parameters. For instance, a previous study [9] showed that the Neo-Hookean model remains unaffected by calibration, and the Arruda–Boyce model is reported to work sufficiently with a single loading mode [10]. A Gaussian treatment of chain deformation leads to the Neo-Hookean model, whereas the eight-chain model recognizes the non-Gaussian nature of chain finite deformation at increasing strain by implementing Langevin statistics [10]. This accounts for the fact that real polymers do not deform in an affine manner due to topological constraints and inherent heterogeneity. This manifests through the observed strain-stiffening visible in Figure 1, which is attributed to structural packing and macromolecular physical rearrangements. The Neo-Hookean model resolves this effect by providing a higher shear modulus and thus a higher number density of network strands than the Arruda–Boyce model, where strain-stiffening is innate in the description of chain deformation. Respectively, the treatment of chain deformation in each model renders the Neo-Hookean model more suitable up to a limited strain before the onset of strain-stiffening, whereas the Arruda–Boyce non-Gaussian depiction marginalizes the affine deformation at small strains [6]. Furthermore, the obtained general scaling (see Figure 2a) $G/G_{0}\approx Q_{v}^{-1/3}$, while considering isotropic conditions, $G∼Q_{v}^{-1/3}∼\phi_{0}^{2/3}\phi^{1/3}$, allows us to assume a theta-solvent [11], which makes it possible to rely on the Gaussian ideal chain model to further determine the mesh size.

As can be seen in Figure 2b, the non-linear elastic response of the hydrogels to loading depends not only on the polymer concentration but is also determined by various structure interaction phenomena, including the binding behavior of the biopolymers towards water molecules. For instance, alginate is hydrophilic in nature owing to the presence of water-solubilizing functional groups, namely -OOC^−^ and -OH. In addition, gelatin contains, on average, eight amino acids (AAs) with an approximately 2:1 ratio of apolar to polar amino acids [12]. This dominant hydrophilicity is the underlying reason for the high swelling capacity of such hydrogels and their viscous behavior. A coupled effect of cross-linking and water content alters the mechanical performance of hydrogels. Bound water is characterized by a higher density [13] in comparison to free water, due to the reduced intermolecular space between the molecules, which contributes to their viscous resistance by increasing the intermolecular forces. Moreover, bound water happens to have higher binding energy and a lower molecular motility [13] that is heightened with increasing cross-linking. Altogether, it raises the internal frictional forces between and within the water phase layers, leading to higher energy dissipation and thus increased hysteresis. The partial oxidation of alginate by cleavage of vicinal diols mainly affects the G-monomeric units [14], thus reducing its affinity to Ca^2+^ and its molecular weight [15], which explains that algGEL_1_ has an initially higher compressive strength than ADAGEL_1_. Schiff’s base formation in ADAGEL_1_ further improves the binding between the co-polymer pairs, which helps to obstruct the network and water phase dislocation under loading by transforming the kinetic energy into a potential form, depicted in higher hysteresis during unloading (Table A1). Selective covalent cross-linking in ADAGEL_2_, along with the absence of physical cross-linking and the presence of hydrophobic network segments, promotes the development of intermingling heterogeneous phases with dense and sparse regions with different water content. This is expressed in a decreasing hysteresis, combined with a slightly increasing compressive strength. Similar results [16] emphasize the role of phase separation in producing hydrogels with high strength and low hysteresis.

The mechanical behavior is susceptible to the amount of absorbed water when placed in a solvent. It can be deduced from Figure 3a that the variations in swelling rates and ratios between the specimens can mainly be attributed to the effect of cross-linking. In algGEL_0_ and algGEL_1_, gelatin and alginate form non-covalent intermolecular interactions. The abundance of carboxylate, amino and carboxyl groups in alginate and gelatin enables the formation of hydrogen bonds. Moreover, the electrical charge on the polymer’s network backbone of alginate (mainly negatively charged carboxylate groups) and gelatin (positively charged molecules due to the protonated amino groups, caused by the acid treatment of collagens to obtain gelatin type A), lead to an electrostatic attraction between them. This in turn causes, a partial charge neutralization which stabilizes the co-polymers’ aggregation [17] without preventing the infiltration of solvent molecules. The partial oxidation of alginate produces aldehyde derivatives that promote Schiff’s base formation [18] through their interaction with the ϵ-amino groups of lysine or hydroxylysine of gelatin. Schiff’s base formation contributes to the structural integrity of ADA-GELs by establishing covalent binding between ADA and gelatin, thus restricting the solubility of polar molecules in the co-polymer network. Moreover, alginate is a polysaccharide that consists of M- and G-monomers arranged in blocks with random sequences. Ionic cross-linking with Ca^2+^ acts on the G-blocks by forming egg-box multimers in a 3D configuration that, along with alginate’s linear structure, exerts a confining effect on its surroundings. And since the alginate (or ADA) was placed in a 0.1 M CaCl_2_ solution, Ca^2+^ ions would diffuse into the hydrogels as they imbibe water and raise the cross-link density within the hydrogels over time, which might explain their de-swelling behavior [19]. This effect was not observed in specimens with ionic cross-linking applied in situ, mainly ADAGEL_4_, which did not undergo further covalent cross-linking like ADAGEL_5_. Gelatin is a polypeptide with an irregular spatial arrangement between its amino acid (AA) groups, which causes unpredictable interaction patterns between them, especially when cross-linking is applied. In this case, mTG acts on the lysine and glutamine amino groups, forming ϵ(γ-glutamyl)lysine isopetide covalent bonds [18]; this decreases the network chains’ diffusion relative to each other, thus contributing to their resistance to swelling. The interaction between water molecules and polymer chain segments is restricted only in the vicinity of these isopeptide bonds. Unless further ionic cross-linking is applied, such as in the case of ADAGEL_3_, swelling would only be locally reduced.

By means of gas pycnometry, we were able to determine the volumetric swelling ratio and densities of the hydrogels, as well as of their polymeric and viscous fractions. Consequently, the properties of interstitial water were acknowledged quantitatively from macroscopic measurements. The hydrogels’ viscous phases with a density and number density higher than that of free water indicate the presence of densely packed water molecules designated as bound water. The hydrophilic functional groups in the co-polymers attract water molecules, which in turn form further hydrogen bonds to adjacent H_2_O. Electrostatic interactions and electrical charge effects, due to the polarity of the functional groups, allow the hydrogels’ network to form a phase with gradual layers of water molecules [20]. The stacked water molecules interacting directly with the polar groups form the strong or primary bound water, also labeled the hydration shell [21], whereas the hydrogen bonds between the primary bound water, as well as interactions between the polymer backbone, and further water molecules form weaker or secondary bound-water areas. As the distance from the polymer’s backbone increases, pockets or pores of free water may form. The hydration shell might actually be acting as a coating that hinders the total solubility of the polymers in a solvent [13]. For instance, and if we omit degradation, when placed in a good solvent, the non cross-linked hydrogels, algGEL_0_ and ADAGEL_0_, tend to swell continuously and indefinitely until the onset of fracture. This is corroborated by the obtained polymer–solvent interaction parameter (see Table 1) as it fulfills 0.5≤χ≤0.55, which indicates that these hydrogels are no longer soluble following polymerization and gelation. A thermal treatment to counteract such an irreversible sol–gel transition can be detrimental to the polymer network structures and functions [22].

Water absorption and permeation occur simultaneously throughout the hydrogel until it reaches an equilibrium state. The polymer–water friction coefficient is defined from Darcy’s law as [23] f=ΔP/vd, where ΔP, *d*, and *v* are the pressure difference, end-to-end flow distance, and the fluid velocity within the polymer network, respectively. The latter is dependent on the swelling ratio and swelling rate and increases as the strands become saturated. This can be viewed in light of fluid permeation in contact with primary or secondary bound water. In [24], f was defined as $f\approx \eta \xi^{-2}$, where η is the dynamic viscosity of the interstitial fluid and ξ is the pore size of a concentration blob. Since the Neo-Hookean rubber elasticity model delivers an average estimate of the mesh size, we can hypothesize that these characteristic lengths are equivalent. f can hence be regarded as a quantifier of the macromolecular physical interaction between the solvent and polymer. We were able to note this as hysteresis was found to increase with f rather than with water content (Figure 4c), and f displays a logarithmic dependence on $n_{M}$ (Figure 4d).

A shortcoming of subjecting the same specimens to mechanical testing, swelling, and further measurements is the early onset of degradation, which was visible by the second and third day. Moreover, a major limitation of this study was to neglect the effect of molecular weight, especially between alginate and ADA, and solely focus on cross-linking as the major influencing factor of both swelling and mechanical behavior. We even skipped determining the molecular weight of the primary chain and between cross-links using the equilibrium-swelling theory, since it is known to provide an over-estimation of these parameters [25]. Although we recognize that these models are idealistic and neglect the inherent heterogeneities of such hydrogels, a good estimation of chains’ molecular weights can be useful in predicting the density of the micro-environment and, thus, the physical confinement exerted on an embedded cell. Moreover, we suggest that a swelling or culturing medium containing ionic cross-linking agents can be implemented as a way of tweaking scaffolds’ properties by alternating the cross-linking density. This can be achieved by exploiting the biomimetic properties of hydrogels by relating a controlled release (application in situ) to degradation or by absorption during swelling (alternating medium concentration of the ions). For instance, Ca^2+^ or Mg^2+^ can exert a dual effect. On the one hand, they cause physical cross-linking in alginate (and ADA) [19,26]; on the other hand, they contribute to chondrocytes’ homeostasis and proliferation [27,28,29,30].

Hydrogels are often perceived as biphasic constructs with elastic and viscous phases, where the interstitial fluid occupies the pore space. However, the hydrophilic nature of biopolymers allows them to form bound water with certain molecular structural aspects, dependent on its intermolecular interaction with the polymer chemical structure [31] and, in synergy with cross-linking and the polymer network, significantly correlate to the hydrogels’ physical properties. That implies that the non-linear elastic mechanical response is dependent on a viscous contribution and on the intertwinement between both phases due to intermolecular interactions.

## 3. Conclusions

In this work, we examined certain dependencies between the macroscopic strain, swelling behavior, and structural parameters of the underlying macromolecular structures, using the bulk and intrinsic properties of the investigated specimens and by employing the rubber elasticity and equilibrium-swelling theories in their classical forms. Despite the idealistic assumptions of the modeling, we managed to predict the average mesh size of the hydrogels’ polymer network and examine it as a characteristic length size related to the water-binding property of the hydrophilic network strands. We thus find that this structural parameter, which was primarily derived from the elastic response, is also an indicator of visco-elasticity through the total energy dissipation. In a future work, we pursue the modeling by overcoming ideal network models, with a focus on degradation kinetics and their effect on diffusive properties within a selection of the studied hydrogels.

## 4. Materials and Methods

### 4.1. Hydrogel Specimens

Alginate-gelatin (alg-GEL) hydrogels and enhanced derivative alginate di-aldehyde gelatin hydrogels (ADA-GEL) were selected due to their established biomimetic properties [14,32,33]. The Alg-GEL and ADA-GEL hydrogels were both prepared using gelatin (GEL) type A (from porcine skin, Bloom 300, Sigma Aldrich, Taufkirchen, Germany). In addition, Alg-GEL was prepared with alginate (VIVAPHARM alginate PH176, JRS Pharma GmbH & Co. KG, Rosenberg, Germany), while oxidized alginate (ADA) (prepared as in [18]) was used for the manufacture of ADA-GEL specimens. For the preparation of the ADA-GELs, we followed the protocol published in [34], which is briefly explained in the following. The gelatin was pre-treated for 3 h at 80 °C under stirring in ultra-pure water (UPW), or in 0.1 M CaCl_2_ (for ADAGEL_4_ and ADAGEL_5_), with an initial concentration of 15.00% (*w*/*w*). Alg, or ADA, was dissolved in Dulbecco’s Phosphate-Buffered Saline (DPBS, Pan Biotech GmbH, Aidenbach, Germany) with an initial concentration of 7.5% (*w*/*w*). The final concentrations of alg (or ADA) and GEL were, respectively, 3.75% and 7.5%*w*/*w* with 1:1 solution ratios. The precursor solutions were stirred for 10 min at 250 rpm at 37 °C to ensure physical blending before either being pipetted into the molds, or in situ addition of enzymatic cross-linking agents. In situ enzymatic cross-linking was performed using microbial transglutaminase (mTG) (Ajinomoto Foods Europe, Hamburg, Germany), prepared as a 50% *w*/*v* solution in DPBS, with an estimated enzymatic activity of 7.5 U/g [34], and added prior to gelation. The reversible covalent cross-linking occurs through Schiff’s base formation between gelatin and ADA due to the formed aldehyde groups in the latter. The sepcimens’ designation due to variations in the cross-linking is summarized in Table 2.

### 4.2. Swelling Experiment

The hydrogel specimens were placed in well-plates in the swelling medium UPW or 0.1 M CaCl_2_ (see Table 2) at 20 °C for 72 h. Their initial and swollen volumes were measured at t_0_ = 0 and then every 24 h using gas pycnometry (Anton-Paar Gaspycnometer Ultrapyc 5000 Foam, Anton-Paar GmbH, Graz, Austria) at a pressure equal to 3 Psi (0.207 bar). The hydrogels’ densities; $\rho_{h}$; mass and volume swelling ratios, respectively, $Q_{m}=100\cdot \left(m_{t}-m_{0}\right)/m_{0}$ and $Q_{v}=100\cdot V_{t}/V_{0}$, were determined. The swelling data were fitted to first-order kinetic $Q\left(t\right)=Q_{eq}\cdot \left(1-e^{-t/\tau }\right)$ in order to predict equilibrium swelling while neglecting degradation and fracture. $V_{0}$ and $m_{0}$ represent the initial volume and mass, while $V_{t}$ and $m_{t}$ are the measured volume and measured mass during swelling. τ is the swelling rate.

#### 4.2.1. Components’ Contents and Average Densities

The hydrogels prepared from the same precursor solutions were dried at room temperature for several days until no further mass change was observed. The average densities of the polymers $\rho_{p}$ as well as the masses and volumes were measured after drying. The initial mass and volumetric fractions $\phi_{p,0}$ of the polymeric component were then determined. Knowing that $\rho_{h}=\sum \rho_{i}\phi_{i}$ (and $\frac{1}{\rho_{h}}=\sum \omega_{i}\rho_{i}$) enabled us to establish the total water contents and corresponding viscous phase density $\rho_{s}$ and the evolution of polymer volumetric fractions $\phi_{p}$ in the as-prepared state and during swelling. $\rho_{h}$ is the hydrogel’s density; $\phi_{i}$ and $\omega_{i}$ are the volume and mass fraction of the viscous or polymer phase. (In this work, we designated the totality of water content within the hydrogels as bulk water.)

#### 4.2.2. Molar Properties of the Interstitial Fluid Phase

Since degradation was neglected, the number of moles $n_{p}$ of the polymer remains constant throughout swelling, while the number of solvent molecules $n_{s}$ changes. By determining the densities $\rho_{s}$ of the viscous phase, at each stage, the average solvent molar volume $V_{1}=M_{m}/\rho_{s}$ and its corresponding average molecular number density $n_{M}=\frac{N_{A}}{M_{m}}\rho_{s}$ can be estimated, where $N_{A}$ is the Avogadro number and $M_{m}$ is the molar mass of water.

### 4.3. Unconfined Constant Strain-Rate Cyclic Compression Tests

The compression tests were performed using a multi-drive rheometer (Anton-Paar multi-drive Rheometer MCR 702, Anton-Paar GmbH, Germany) at 20 °C using a parallel-plate setup. The hydrogels were characterized in their as-prepared state and following swelling in medium every ∼24 h up to 3 days. The specimens underwent cyclic compression for 5 cycles at a constant deformation rate of 300 $µm\;s^{-1}$ up to ∼20% strain, with a static pre-load of 0.05 ± 0.01 N to ensure constant and complete plate–specimen contact. A one-minute recovery period was applied between each cycle in order to neglect visco-elastic conditioning and minimize time-dependent effects such as stress-relaxation and creep. The specimens were not glued, and device settings secured full and continuous contact with the specimens during testing. The obtained responses were used for an analytical hyperelastic materials’ parameter identification. We evaluated the experimental results for fitting to the materials’ models using Drucker’s first stability criterion to ensure monotonically non-decreasing stress–strain responses by verifying the slope incrementally, $\partial \sigma_{i}/\partial \epsilon_{i}⩾0$.

### 4.4. Polymer Average Structural Parameters

#### 4.4.1. Molecular Models of Hyperelastic Strain Energy Density Function

The rubber elasticity theory describes finite macro-scale deformation through statistical mechanics starting from the deformation of a single chain, in terms of entropy elasticity, $s=k_{B}\ln\left(\Omega \left(N,r\right)\right)$, where $k_{B}$ is the Boltzmann constant and Ω the number of possible conformations of a single chain model with *N* segments and end-to-end vector r=Nl. We applied two hyperelastic constitutive models where deformation is defined only by the first invariant, $I_{1}=\lambda_{1}^{2}+\lambda_{2}^{2}+\lambda_{3}^{2}$, in terms of the principle stretches $\lambda_{i},i=1,2,3$. For uniaxial deformation and under the assumption of isotropy and incompressibility, $\lambda_{1}=\lambda$, $\lambda_{2}=\lambda_{3}=\lambda^{-1/2}$, and $I_{1}=\lambda^{2}+2\lambda^{-1}$. The hyperelastic models are concisely outlined.

##### Neo-Hookean Model

The nominal stress σ of uniaxial deformation is the applied axial force *f* divided by the specimen’s area $a_{0}^{2}$. The deforming force is obtained by differentiating the change in free energy $\Delta F=-T\sum^{n}\Delta s=\frac{nk_{B}T}{2}\left(\lambda^{2}-2\lambda^{-1}-3\right)$, for *n* network strands, by the main deformation length $\lambda a_{0}$, such as $f=\frac{\partial \Delta F}{\partial \lambda a_{0}}=\frac{nk_{B}T}{a_{0}}\left(\lambda -\lambda^{-2}\right)$, and where *s* is the entropy elasticity of an ideal Gaussian chain:

(1) $$s\left(N,r\right)=-\frac{3k_{B}Tr^{2}}{2Nl^{2}}+s\left(N,0\right)$$

The Neo-Hookean uniaxial stress–strain response is as follows:

(2) $$\Rightarrow \sigma =\frac{f}{a_{0}^{3}}=\frac{nk_{B}T}{V}\left(\lambda -\lambda^{-2}\right)=\nu k_{B}T\left(\lambda -\lambda^{-2}\right)$$

The modulus of rigidity of an affine network is $G_{\mathrm{NH}}=\nu k_{B}T$, with ν being the number density of elastically active network strands.

##### Arruda–Boyce 8-Chain Model

The Arruda–Boyce model defines the network strands of length $r_{0}=\sqrt{N}l$ on the diagonals of an elementary unit cubic cell of dimension $\alpha_{0}$. Following deformation, $r_{\mathrm{chain}}=r_{0}\lambda_{\mathrm{chain}}=\frac{\alpha_{0}}{2}{\left(\lambda_{1}^{2}+\lambda_{2}^{2}+\lambda_{3}^{3}\right)}^{1/2}$, with $\lambda_{\mathrm{chain}}=\frac{1}{\sqrt{3}}I_{1}^{1/2}$. The elastic entropy of a deformed chain is described in Equation 3 using inverse Langevin function $\beta =L^{-1}\left[\lambda_{\mathrm{chain}}N^{-1/2}\right]$ that takes finite chain deformability into consideration, such as $\lambda_{chain}\to \lambda_{l}$, where $\lambda_{l}=\sqrt{N}$ is limiting or locking stretch.

(3) $$s\left(N,r_{\mathrm{chain}}\right)=c-k_{B}T\left(\frac{\lambda_{\mathrm{chain}}}{\sqrt{N}}\beta +\ln\left(\frac{\beta }{\sinh\beta }\right)\right)$$

Similarly, the non-Gaussian force–deformation relationship is obtained by differentiating the entropy change function −TΔs over $r_{\mathrm{chain}}$ yielding $f=\frac{k_{B}T}{l}L^{-1}\left[\frac{r_{\mathrm{chain}}}{r_{l}}\right]$. For uniaxial deformation, the total entropy change per unit volume with a total of ν chain density is $\Delta S=\frac{\nu }{3}\Delta s$. The nominal stress is thus:

(4) $$\sigma_{8c}=\frac{\partial \Delta F}{\partial \lambda_{chain}}=\frac{\nu k_{B}T}{3}NL^{-1}\left[\frac{\lambda_{chain}}{\lambda_{l}}\right]\left(\frac{\lambda^{2}-\lambda^{-1}}{\lambda_{\mathrm{chain}}}\right)$$

In this case, $\frac{\nu k_{B}T}{3}$ represents the shear modulus $G_{\mathrm{AB}}$.

Using Matlab (MathWorks, Inc. (2021) MATLAB version: 9.11.0 (R2021b)), the stress–strain responses from Section 4.3 were fitted to both models, Equations (2) and (4), using the non-linear least-square method. The inverse Langevin function was computed using Petrosyan approximation for a $L^{-1}\left[f\left(x\right)\right]$ since it is valid for x≥0, with a relative error bound of the order of $10^{-3}$ [35,36].

#### 4.4.2. Characteristic Length of the Polymer Network

For estimating the average mesh size ξ[L] and cross-linkage number density *c*$\left[L^{-3}\right]$, we rely on a simplified representative cubic element within an ideal perfect 3D network model with homogeneous cross-linking density, such as c=ν/2, where the average mesh size represents the length of a network strand and the distance between two cross-linkages, $\xi ={\left(1/c\right)}^{1/3}$.

The hyperelastic models are based on the assumption of no variation in the internal energy, ΔU=0, that is otherwise present in non-linear visco-elastic deformation. Nevertheless, we examined the obtained structural parameters in relation to hysteresis. Hysteresis is the total dissipated energy in each loading–unloading cycle, $W_{\mathrm{diss}}=\Delta U+\Delta Q$, with ΔQ being the thermal energy dissipation. Here, we consider the blob size and the obtained ξ to be identical and observe hysteresis in relation to the friction coefficient, f ($N\;s\;m^{-4}$), between the polymer network and the imbibed interstitial fluid, which is, according to [24]$,f\propto \rho_{s}\xi^{-2}$, where the blob size represents a concentration-dependent characteristic length.

#### 4.4.3. Equilibrium Swelling

Rubber elasticity characterizes the deformation of an isotropically swollen network by assuming $\lambda ={\left(\frac{Q_{v}}{100}\right)}^{1/3}={\left(\frac{\phi_{p,0}}{\phi_{p}}\right)}^{1/3}$[5,7]. Polymer network swelling is described by a change in Gibbs free energy $\Delta G_{mix}$ due to configurational entropy alterations caused by both polymer–solvent mixing and their influence on possible strands’ conformations. This can be described in terms of the chemical potentials Δμ of elastic and swelling free energies by considering molecular compositional changes:

(5) $$-\frac{N_{A}}{V_{1}}\left({\left(\frac{\partial \Delta G_{mix}}{\partial n_{s}}\right)}_{n_{p},T,P}+{\left(\frac{\partial \Delta G_{el}}{\partial n_{s}}\right)}_{n_{p},T,P}\right)\overset{\mathrm{equil}.}{=}0$$

With:

(6) $$\begin{array}{cc} \Delta \mu_{mix}={\left(\frac{\partial \Delta G_{mix}}{\partial n_{s}}\right)}_{n_{p},T,P} & =-\frac{N_{A}k_{B}T}{V_{1}}\left(\phi_{p}+\ln\left(1-\phi_{p}\right)+\chi \phi_{p}^{2}\right)\\ \Delta \mu_{el}={\left(\frac{\partial \Delta G_{el}}{\partial n_{s}}\right)}_{n_{p},T,P} & =\nu_{0}N_{A}k_{B}T\left(\frac{1}{2}\lambda^{-3}-\lambda^{-1}\right) \end{array}$$

Thus, an affine perfect polymer network at swelling-equilibrium is as follows:

(7) $$-\frac{k_{B}T}{V_{1}}\left(\phi_{p,eq}+\ln\left(1-\phi_{p,eq}\right)+\chi \phi_{p,eq}^{2}\right)+\nu_{0}k_{B}T\left(\frac{1}{2}\left(\frac{\phi_{p,eq}}{\phi_{p,0}}\right)-{\left(\frac{\phi_{p,eq}}{\phi_{p,0}}\right)}^{1/3}\right)=0$$

From the applied fit in Section 4.2, the equilibrium volumetric swelling ratio, $Q_{v,eq}$, and the equilibrium parameters, $\phi_{p,eq}$ and $V_{1_{eq}}$, were predicted. The polymer–solvent interaction parameter χ for each hydrogel group was thus determined.

## Figures and Tables

**Table 1:** Average structural parameters obtained in initial and from swelling-equilibrium states.

Sample Designations	Average Parameters						
	*Q_v,eq_*	*ϕ_p,0_*	*ϕ_p,eq_*	$\nu_{0_{NH}}$ *	$\nu_{0_{AB}}$ *	$V_{1_{\mathrm{eq}}}$	χ
	(%)	[1]	[1]	$\cdot 10^{6}$ ${µm}^{-3}$	$\cdot 10^{6}$ ${µm}^{-3}$	${\mathrm{cm}}^{3}$ ${\mathrm{mol}}^{-1}$	[-]
algGEL_0_	2000	0.182	0.008	4.030	0.703	17.777	0.504
algGEL_1_	235	0.155	0.077	4.970	0.664	17.755	0.527
ADAGEL_0_	2421	0.134	0.006	3.160	0.827	17.777	0.505
ADAGEL_1_	220	0.137	0.063	2.915	1.006	17.759	0.522
ADAGEL_2_	776	0.140	0.018	4.081	0.566	17.773	0.507
ADAGEL_3_	100	0.135	0.133	3.943	1.031	17.737	0.550
ADAGEL_4_	417	0.134	0.030	1.812	1.556	17.770	0.510
ADAGEL_5_	169	0.126	0.084	3.265	1.156	17.753	0.530

*: initial average number density of elastically effective chains.

**Table 2:** Variations in the cross-linking and specimens.

Sample Designations	Cross-Linking *			Swelling Medium
	0.1 M CaCl_2_	50% *w*/*v* mTG (In Situ) Enzymatic Activity ∼ 7.5 U/g	Schiff’s Base Formation Due to Formed Aldehyde Groups	
	Ionic	Covalent	Reversible Covalent	
algGEL_0_	-	-	-	UPW
algGEL_1_	*√*	-	-	0.1 M CaCl_2_
ADAGEL_0_	-	-	*√*	UPW
ADAGEL_1_	*√*	-	*√*	0.1 M CaCl_2_
ADAGEL_2_	-	*√*	*√*	UPW
ADAGEL_3_	*√*	*√*	*√*	0.1 M CaCl_2_
ADAGEL_4_	*√* (in situ)	-	*√*	UPW
ADAGEL_5_	*√* (in situ)	*√*	*√*	UPW

*: (*√*) applied cross-linking.

## Data Availability

Further data are included in the Table A1. Raw and further data can be provided upon request from the corresponding author (N.A.).

## Appendix A

**Table A1:** Average parameters.

Samples	Time (d)	$Q_{v}$ (-)	$\phi_{p}$ (%)	$G_{\mathrm{NH}}$ (kPa)	$G_{\mathrm{AB}}$ (kPa)	ξ (nm)	$\rho_{h}$ ($g\;{\mathrm{mL}}^{-1}$)	$\rho_{s}$ ($g\;{\mathrm{mL}}^{-1}$)	W_diss_ ($\mathrm{mJ}\;{\mathrm{mm}}^{-3}$)
algGEl_0_	0		15.49±1.23	16.36	1.57	7.9	1.337±0.024	1.369±0.042	0.500±0.066
	1	9.55	1.82±0.31	4.43	0.31	12.2	1.046±0.016	1.042±0.014	0.038±0.007
algGEL_1_	0		18.16±1.42	20.18	2.11	7.4	1.474±0.109	1.549±0.140	0.542±0.004
	1	2	7.72±0.57	20.17	1.66	7.4	1.218±0.009	1.224±0.010	0.192±0.047
	2	1.9	7.83±0.36	10.27	1.05	9.2	1.190±0.005	1.205±0.005	0.12±0.018
	3	1.74	7.34±0.51	10.42	1.06	9.2	1.242±0.023	1.250±0.026	0.124±0.011
ADAGEL_0_	0		13.43±1.01	12.83	0.95	8.6	1.327±0.032	0.150±0.044	0.150±0.044
	1	5.15	2.07±0.36	7.65	0.56	10.2	1.051±0.012	1.051±0.012	0.043±0.001
	2	8.6	1.33±0.13	7.00	0.6	10.5	1.026±0.002	1.026±0.002	0.048±0.005
	3	11.9	0.98±0.09	5.57	0.46	11.3	1.012±0.002	1.012±0.002	0.047±0.005
ADAGEL_1_	0		13.74±0.12	11.84	0.90	8.9	1.336±0.028	1.353±0.018	0.123±0.045
	1	2.05	6.62±0.03	15.02	0.99	8.1	1.125±0.018	1.156±0.009	0.176±0.036
	2	1.97	6.81±0.07	15.94	1.20	8.0	1.094±0.010	1.157±0.004	0.220±0.037
	3	2	6.66±0.10	14.97	1.09	8.2	1.062±0.005	1.156±0.005	0.157±0.025
ADAGEL_2_	0		14.02±0.95	16.57	1.12	7.9	1.336±0.028	1.350±0.034	0.106±0.001
	1	2.7	6.10±0.86	11.96	0.98	8.8	1.125±0.018	1.117±0.017	0.037±0.003
	2	3.86	4.02±0.40	13.92	1.32	8.4	1.094±0.010	1.087±0.010	0.043±0.003
	3	4.64	3.26±0.34	10.69	1.08	9.1	1.062±0.005	1.056±0.005	0.035±0.002
ADAGEL_3_	0		13.35±1.73	7.36	1.36	8.0	1.280±0.066	1.250±0.073	0.136±0.009
	1	0.92	11.59±1.37	2.93	2.1	7.0	1.246±0.016	1.215±0.014	0.045±0.006
	2	0.72	10.42±1.48	2.29	2.63	6.5	1.334±0.035	1.371±0.044	0.036±0.003
	3	0.59	8.51±1.13	3.13	2.01	7.0	1.426±0.076	1.421±0.083	0.044±0.007
ADAGEL_4_	0		13.37±0.48	13.26	0.77	10.3	1.270±0.01	1.244±0.011	0.200±0.022
	1	3.94	3.46±0.06	18.94	0.29	14.0	1.083±0.004	1.077±0.007	0.045±0.006
	2	3.81	3.52±0.26	18.31	0.24	15.3	1.079±0.015	1.07±0.015	0.036±0.003
	3	3.62	3.71±0.26	23.40	0.25	13.7	1.095±0.016	1.081±0.016	0.044±0.007
ADAGEL_5_	0		12.45±0.14	13.26	1.4	8.5	1.220±0.006	1.200±0.006	0.229±0.007
	1	1.77	7.03±0.18	18.94	2.35	7.5	1.160±0.01	1.14±0.01	0.264±0.053
	2	1.65	7.55±0.14	18.31	2.38	7.6	1.137±0.004	1.114±0.004	0.239±0.033
	3	1.44	8.65±0.15	23.4	2.11	7.0	1.195±0.004	1.173±0.004	0.210±0.017
